# Is practice good enough? Retrieval benefits students with ADHD but does not compensate for poor encoding in unmedicated students

**DOI:** 10.3389/fpsyg.2023.1186566

**Published:** 2023-07-20

**Authors:** Meredith E. Minear, Jennifer H. Coane, Leah H. Cooney, Sarah C. Boland, Judah W. Serrano

**Affiliations:** ^1^Department of Psychology, University of Wyoming, Laramie, WY, United States; ^2^Department of Psychology, Colby College, Waterville, ME, United States

**Keywords:** memory, ADHD, testing effect, retrieval practice, college students

## Abstract

**Introduction:**

A significant proportion of currently enrolled college students receive support for attention deficit/hyperactivity disorder (ADHD) and these students are often at risk of academic failure. Retrieval practice or self-testing is an effective, accessible, and affordable tool for improving academic performance. Three recent studies found conflicting results with regards to the effectiveness of retrieval practice in this population.

**Methods:**

The present study compared 36 individuals with ADHD to 36 controls. Participants studied Swahili-English word pairs that varied in difficulty. Half of the pairs were repeatedly studied, and the other half repeatedly tested.

**Results:**

On a final test, all participants showed a benefit of retrieval practice relative to restudy and participant status did not moderate the effect. However, unmedicated individuals with ADHD performed worse overall, both during the encoding phase and on the final test, whereas medicated participants were not significantly different from controls.

**Discussion:**

An examination of self-reported encoding strategies found unmedicated participants used fewer deep strategies at encoding, consistent with prior work on ADHD and memory. Although retrieval practice is effective in this group, improved strategy use may be necessary to ensure performance that is fully equivalent to that of students without ADHD.

## Introduction

Attention deficit/hyperactivity disorder (ADHD) is characterized by inattentiveness and/or hyperactivity/impulsivity, high levels of distractibility, difficulty maintaining focus and sustaining attention (Barkley, 1997). Initially described as a developmental disorder, approximately 2/3rds of children with ADHD have symptoms persisting into adulthood (Resnick, 2005; Sibley et al., 2022). A growing number of these young adults pursue higher education, constituting an estimated 2% to 8% of the college population (Raue and Lewis, 2011). Unfortunately, college students with ADHD are more likely to drop out, have lower grade point averages (GPA), and report lower academic self-efficacy than students without a diagnosis (Heiligenstein et al., 1999; Weyandt and DuPaul, 2008). Although the effects of ADHD on academic performance are relatively understudied in college students compared to children, both cognitive and non-cognitive factors have been proposed as contributing to these students’ difficulties in adjusting to college (Weyandt et al., 2013; Jarrett, 2016).

Long-term memory is important for academic success, and adults with ADHD perform worse on tests of verbal long-term memory (Hervey et al., 2004; Skodzik et al., 2017). Two sources of poor memory performance are failures to encode the information during the initial study phase and failures to retrieve stored information at test. In ADHD, memory deficits appear to be mediated largely by poor encoding (Skodzik et al., 2017) due to the use of less effortful learning strategies (Reaser et al., 2007; Egeland et al., 2010; Knouse et al., 2012). The importance of learning strategies has been well-studied and demonstrated in multiple studies (Dunlosky et al., 2013). Low effort or shallow encoding strategies typically focus on the surface features of a stimulus such as shape or sound. For example, highlighting a piece of text or simply repeating a word over and over to remember it would be examples of shallow processing. Conversely, deep encoding methods focus on meaning such as creating a visualization of the information to be learned, forming a sentence with a word to be learning or tying new information to previous knowledge (Craik and Tulving, 1975).

Another strategy that has been shown to improve long-term retention is the act of testing oneself during encoding also known as retrieval practice (Roediger and Butler, 2011; Dunlosky et al., 2013). This mnemonic benefit is referred to as the *testing effect* (TE) and has been found across different types of materials and different populations (Coane, 2013; Rowland, 2014; Karpicke et al., 2016).

Self-testing has the potential to specifically benefit individuals with ADHD as there is evidence that learners with poorer strategies benefit more from retrieval practice than individuals using more effective encoding (Minear et al., 2018; Robey, 2019). Furthermore, because self-testing occurs during the encoding process, it could serve as an effective strategy to mitigate some of the observed encoding difficulties reported (Skodzik et al., 2017). Thus, it is surprising that there are only three studies of the benefits of retrieval practice for college students with ADHD, with one study failing to observe any benefit from retrieval practice in students with ADHD (Dudukovic et al., 2015), and two studies finding that testing yielded similar increases in performance in students with ADHD as in control participants (Knouse et al., 2016, 2020).

Dudukovic et al. (2015) found no benefit of testing for students with ADHD using the free recall of three prose passages. Each passage had 30 possible idea units. After an initial study period, one passage was restudied, a second was tested using free recall, and in a third condition, participants completed math problems with no opportunity for restudy or testing. The control group without ADHD showed greater initial recall of the prose passage compared to the participants with ADHD. At final retrieval, the control group also showed a benefit of testing when comparing testing to the math condition, but not when comparing test to restudy. Thus, even in the control group, the traditional TE (measured relative to a restudy condition) did not occur. However, the group with ADHD showed no benefit of testing regardless of whether the comparison was to restudy or to the math condition. The lack of a TE for individuals with ADHD was attributed to their poor performance on the initial recall task, consistent with evidence that encoding deficits are a key factor influencing memory in this population.

The next study also used free recall as the testing paradigm with 2 lists of 48 words (Knouse et al., 2016). Each list had words from 8 categories with 6 semantically related words per category. One list was restudied 8 times while the other alternated between 4 restudy periods and 4 free recall tests. Control participants were matched to those with ADHD on final performance for words that were studied, but not tested, during the encoding phase. Thus, any group differences in the testing effect would not be a result of differences in scale (Knouse et al., 2016). In contrast to Dudukovic et al. (2015), they found both groups performed equally well during the encoding phase, showing similar increases in performance across study-recall blocks. Furthermore, both groups benefited equally from retrieval practice, recalling more items that had been previously tested than items that were restudied. No differences between participants with ADHD and control participants emerged in a final recognition task or in measures of relational or item-specific processing, either.

In the most recent study, Knouse et al. (2020) compared students with ADHD and those without ADHD under conditions of self-regulated retrieval practice compared to a set criterion of successful retrievals. Given reported deficits in strategy use on the part of students with ADHD (Advokat et al., 2008), they hypothesized that students with ADHD would perform worse under conditions of self-regulation, in which the students determine how frequently they engaged in self-testing. They used 14 key term definitions drawn from short psychology passages as the material to be learned and used both cued recall and recognition of the definitions at final test. However, their hypothesis was not supported as both the group with ADHD and the control group benefited equivalently from retrieval practice whether testing was self-regulated or a function of a set number of successful retrievals.

From these three studies, it appears that college students with ADHD either fail to benefit or benefit equivalently from testing as their non-ADHD peers. However, there is growing appreciation that multiple factors can affect the extent to which individuals can benefit from retrieval practice. In addition to success on the initial retrieval attempt (Dudukovic et al., 2015), greater difficulty or effort involved in retrieval is theorized to be beneficial with a larger testing effect resulting from more difficult tests, such as free recall, over easier recognition tests (Rowland, 2014). Additional difficulty manipulations such as the number of correct retrievals and the spacing of retrieval also produce larger testing effects (Rowland, 2014). However, the evidence is more mixed when difficulty is a function of the type of material being learned. Vaughn et al. (2013) found that more difficult word pairs were retained less well, even when the number of successful recalls during encoding was equated between easy and difficult pairs. de Lima and Jaeger, (2020) found mixed results, with a larger testing benefit for easy items in one experiment and a trend for more difficult items in a second study. Minear et al. (2018) found that the size of the TE based on item difficulty interacted with individual differences in fluid intelligence. Specifically, individuals with low fluid intelligence showed larger testing effects on easy items and individuals high in fluid intelligence benefited more on difficult items. Previous studies of ADHD have not directly addressed the possible role of item difficulty although, across studies, the type and complexity of materials have varied.

In the present study, we compared the benefits of retrieval practice between college students with and without ADHD using a well-studied cued-recall paradigm with Swahili-English word-pairs. Associative memory typically requires more engagement of controlled processes as it requires the binding of cue and target (Naveh-Benjamin, 2000); thus, this task relies more heavily on effortful processing during encoding. We directly manipulated item difficulty so that one set of items was easier to learn and another more difficult. Item difficulty had previously been sensitive to participant differences in fluid intelligence (Minear et al., 2018) and we hypothesized that item difficulty may interact with ADHD status, with group differences emerging when demands on associative memory are greater. However, the directionality of such a difference is uncertain. On the one hand, the use of poorer encoding strategies by individuals with ADHD will likely be less effective for difficult items; thus, they may benefit more from retrieval practice, which would replace the strategies they would spontaneously use. However, it is also possible that the initial retrieval success for difficult items during encoding may be so poor that there will be a smaller TE for difficult items for students with ADHD, similar to those seen in previous studies for individuals with low fluid reasoning (Minear et al., 2018).

As noted, college students with ADHD often face higher risks of failing courses, lower GPAs and dropping out or taking longer to complete their coursework. Stimulant medications can be effective in ameliorating many of the behavioral and cognitive symptoms of ADHD, although they are often insufficient at normalizing performance (Gualtieri and Johnson, 2008). However, even among college students taking medication, performance on core metrics (such as GPA) remained lower than among students without a diagnosis of ADHD (Advokat et al., 2011; see Advokat, 2010, for a review). Among college students with ADHD, however, there is evidence that better study strategies can improve academic outcomes (Advokat et al., 2008). Thus, we include exploratory analyses on the effects of self-reported medication use.

## Materials and methods

### Participants

A total of 72 participants were included in the final data analyses, 36 students with an ADHD diagnosis and a comparison group of students (*n* = 36), selected from a larger sample of over 300 participants who participated in a previous study on the benefits of retrieval practice. Control participants were selected from this pool to match individuals with ADHD in age, biological sex, GPA, and measures of cognitive performance (i.e., fluid intelligence, working memory, and vocabulary; see Table 1). This was done because in a previous study fluid intelligence and vocabulary interacted with item difficulty (Minear et al., 2018), and other studies have reported effects of working memory on the testing effect (Tse and Pu, 2012; Agarwal et al., 2017). Thus, any differences in memory performance or in the magnitude of the testing effect seen between groups cannot be attributed to these variables in this study. We also collected measures of academic self-efficacy, study habits, and test anxiety, but these were not matched between groups.

**Table 1:** Descriptive statistics and cognitive performance of participants with ADHD and control participants.

	ADHD (*n* = 36)	Control (*n* = 36)	*p*-value
Age	19.69 (1.51)	19.69 (1.51)	>0.99
# Women	20	20	n.s.
GPA	3.18 (0.45)	3.27 (0.56)	0.50
Ravens	28.14 (9.59)	27.87 (9.52)	0.94
Operation span	41.17 (18.48)	43.39 (20.16)	0.63
Symmetry span	20.42 (9.96)	18.19 (10.39)	0.36
Shipley vocabulary	29.92 (4.08)	30 (3.69)	0.93
ADHD checklist	20.21 (11.20)	10.39 (6.73)	<0.001
SELF-A	65.19 (13.03)	73.53 (10.98)	<0.01
Test anxiety	68.50 (17.42)	63.44 (16.58)	0.21

SELF-A, Self-efficacy for learning (abridged).

Students with a diagnosis of ADHD were recruited in two ways. Fifteen were referred through the office of the Dean of Students at a small liberal arts college in the northeast (this office provides accommodations for students with learning disabilities and other health issues) and via email, and the remaining 21 students signed up via the participant sign-up pools at the college as well as a state university and self-identified as having a diagnosis. Participants reported the diagnosis was made by a doctoral level therapist in seven cases, by a practitioner holding a master’s degree in two, and by a medical doctor in the remaining cases. Participants were asked to answer yes or no whether they were currently taking medication to treat their ADHD but were not asked to report the type(s) of medication used. Twenty-one participants (58%) reported taking medication at the time of the study and one regularly took medication but did not take it the day of the study. In addition to self-report, all participants, except for two individuals with ADHD, completed a current symptom checklist (Barkley et al., 2008) that measures frequency of behaviors such as difficulty paying attention or listening over the previous 6 months.

We aimed to recruit as many participants as possible, and, at minimum, a sample size for participants with ADHD comparable to previous studies (i.e., 27, as in Knouse et al., 2016) and stopped data collection after four semesters of testing. The Institutional Review Boards at both institutions approved the study. Participation lasted approximately 3 h, over 2 days, and participants were compensated with $30 or course credit.

### Materials

#### Paired associates task

A set of 48 Swahili-English word pairs (e.g., *mbwa*-*dog, ankra-invoice*) were selected from the Nelson and Dunlosky (1994) norms. The norms provide estimates of item difficulty over three consecutive study-test trials. For the study, 12 pairs at each of four levels of difficulty were selected. Based on the norming data, correct recall after the third block was 0.44 (*SD* = 0.05), 0.55 (*SD* = 0.04), 0.72 (*SD* = 0.04), and 0.84 (*SD* = 0.07), from most difficult to easiest, respectively. The 24 pairs drawn from the two easiest levels constituted the Easy set of items and the 24 pairs drawn from the two hardest difficulty levels constituted the Difficult set. Half of the pairs from each difficulty set were assigned to the repeated study condition and half to the repeated test condition with the stimulus condition counterbalanced across participants.

**Figure 1:**
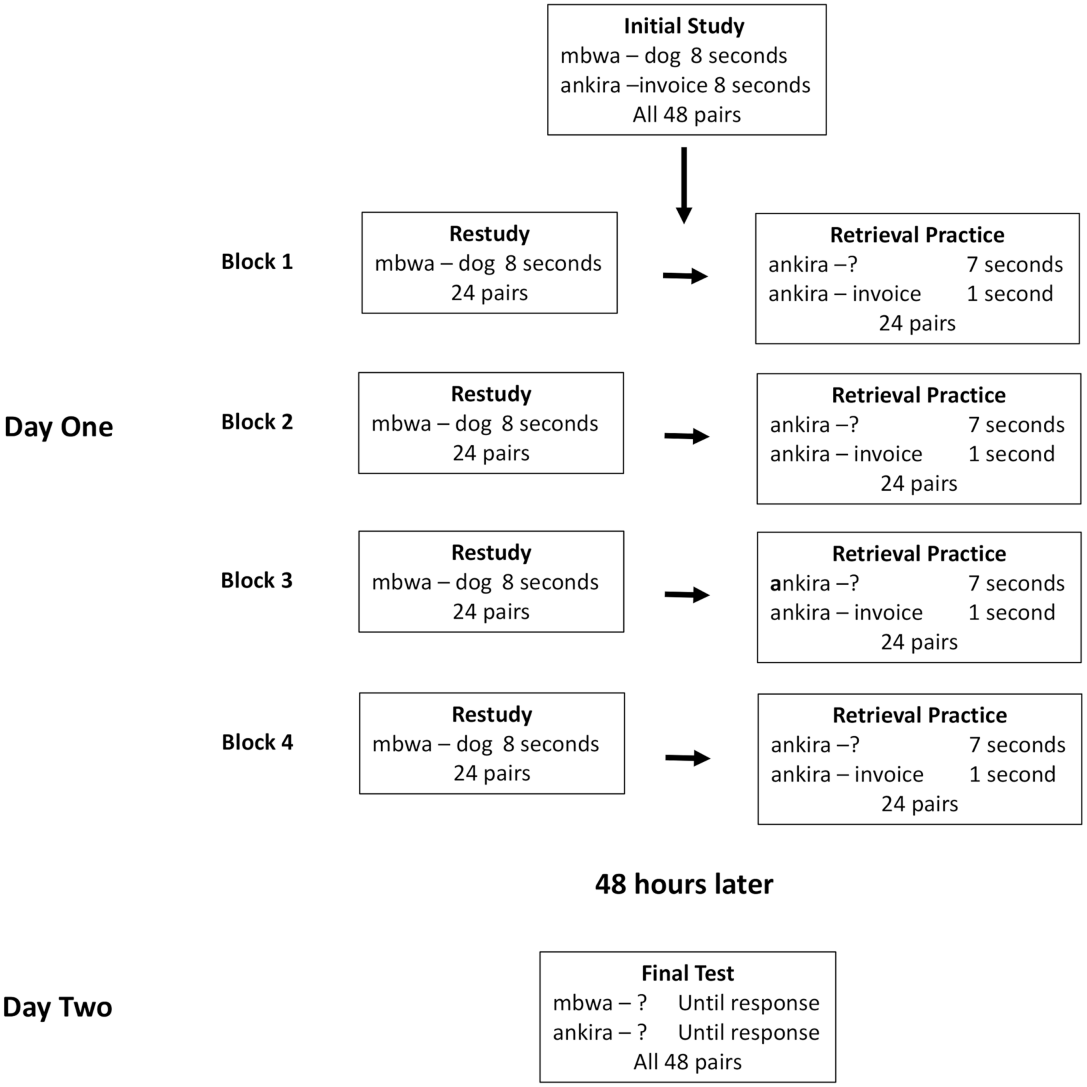
Schematic illustration of the paired-associates task.

Participants first studied all 48 pairs, in random order, for 8 s each. They next completed four additional blocks of repeated study and repeated testing. The re-study block always preceded the repeated test block to avoid contamination from prior retrieval (*cf.* Brewer and Unsworth, 2012). In the re-study blocks, 24 pairs were presented for 8 s each. In the repeated test blocks, the other 24 pairs were tested by having the Swahili cue (e.g., *mbwa*-?) appear for 7 s, during which time participants typed their response using the computer’s keyboard, followed by the intact pair for 1 s (e.g., *mbwa*-*dog*). Thus, the repeated test block included feedback. Pair order within blocks was randomized anew on each block. Responses during the encoding and final test phases were coded as correct if participants recalled the correct answer or minor spelling/morphological variations (e.g., *clouds* instead of *cloud*). See Figure 1 for an overview of the paired associates task.

Two days later, participants returned to the lab and completed a final cued recall test. All 48 Swahili cues were presented, one at a time, in random order. Participants had unlimited time to respond and no feedback was given.

#### Cognitive battery

Participants completed a set of cognitive tasks to measure individual differences in intelligence and working memory. As noted in the Participants section, this task battery was administered to allow us to match participants with ADHD to participants without ADHD on fluid intelligence, working memory and vocabulary.

##### Ravens advanced progressive matrices

We used a computerized version of this measure of general fluid intelligence. On each trial, participants were presented a matrix of eight shapes/complex patterns and had to select one of four options that best completed the matrix across rows and columns. The task was self-paced and consisted of 48 trials of increasing difficulty (Raven et al., 1994).

##### Operation span

We used the automated version of this measure of verbal working memory as developed by Unsworth et al. (2005). Participants were presented a series of 2–7 letters presented sequentially. Between each letter, a simple arithmetic operation was presented, and after indicating whether the equation was true or false, participants indicated which letters had been presented by using the computer mouse to click on the correct letters, in order in which they had been presented. The task consisted of 15 trials of varying length and the scoring was based on correctly recalled letters in the correct order of input.

##### Symmetry span

In this computerized measure of visuospatial working memory, participants had to remember the order in which 2–5 locations lit up on a 4 × 4 grid (Unsworth et al., 2005). Between each trial, a shape was presented and participants had to indicate whether it was symmetrical. Following the symmetry decision, a blank 4 × 4 grid was presented and participants clicked on the correct locations using the mouse. Each participant completed 12 trials.

##### Shipley vocabulary

A computerized version of Shipley (1940) was used to measure vocabulary, a proxy for crystallized intelligence. On each trial, a target word appeared with four options. Participants selected the option that most closely matched the target’s meaning. Items increased in difficulty across trials and higher scores (max = 40) reflect greater vocabulary knowledge.

#### Survey measures

##### ADHD symptom checklist

The measure consists of 18 questions asking participants to indicate how often, in the previous 6 months, they experienced specific behaviors, such as interrupting others, not finishing work, or feeling restless (Barkley et al., 2008). Responses are given on a 0 (*never or rarely*) to 4 (*very often*) scale and higher scores (max = 72) reflect greater severity of symptoms.

##### Study habits

This measure consists of 12 questions assessing how students typically study and is a modification of a survey developed by Kornell and Bjork (2007). Questions assess how individuals schedule their study time, how they decide what to study next, whether they self-test or reread, and what their GPA is (Hartwig and Dunlosky, 2012).

##### Self-efficacy for learning form abridged

This measure contains 19 items asking students to rate their self-efficacy for academic tasks such as reading, writing, studying, taking notes and tests using a scale from 0 (*Definitely cannot do it*) to 100 (*Definitely can do it*; Zimmerman and Kitsantas, 2007) with the average score across items indicating self-efficacy for learning with a higher score corresponding to greater self-efficacy.

##### Cognitive test anxiety scale

This scale consists of 27 questions assessing a student’s anxiety about taking tests and failing tests as well as the number of irrelevant thoughts encountered during tests. Responses are given on a 4-point scale from 0 (*Not at all typical of me*) to 3 (*Very typical of me*) scale with higher scores (max = 81) indicating greater test anxiety (Cassady and Johnson, 2002).

### Procedure

The study took part over 2 days. Each session lasted approximately 90 min. Participants were tested individually. On the first day, after providing consent, participants completed the paired-associates task and Ravens. Two days later, participants first took the final cued recall test on the paired associates, followed by the two working memory tasks and Shipley. They then were given a paper packet that included an open-ended question about what strategy/ies they used to learn the pairs, the ADHD symptom scale, the self-report measures of academic self-efficacy, cognitive test anxiety, demographic information, and a question asking whether they had ever been diagnosed with ADHD (if they answered positively, they were further asked who had made the diagnosis). The tasks were administered in such a manner that the two sessions lasted approximately an equal amount of time (90 min). At the conclusion of the second session, participants were thanked, debriefed, and compensated.

## Results

We begin our results section by first reporting the demographic characteristics of each group. Although we matched participants on fluid intelligence, working memory and vocabulary, we measured other traits, academic self-efficacy and test anxiety, that we expected to differ between groups based on prior ADHD research (Heiligenstein et al., 1999; Nelson et al., 2014; Dan and Raz, 2015). We then proceed to report the initial encoding of the word pairs, whether there were differences between groups in their initial learning and in the encoding strategies participants reported using. Next, we address the final recall data and the crux of our paper, i.e., whether group differences are seen for the TE and whether this depends on item difficulty or any group differences in the demographic measures. In our final section, we examine post-hoc whether the medication status of our participants with ADHD affected our results.

### Demographics

The data from all individual difference measures are reported in Table 1. By design, there were no differences between groups on our measures of fluid intelligence, working memory and vocabulary, all *t* values < 1. As expected, there was a significant difference in reported ADHD symptoms, *t*(68) = −4.47, *p* < 0.001, *d* = 1.1. There was also a significant difference in academic self-efficacy, *t*(70) = 2.94, *p* < 0.01, *d* = 0.69, with lower self-efficacy for learning in our ADHD group which is consistent with prior reports (Heiligenstein et al., 1999), but no difference in reported test anxiety *t*(70) = −1.26, *p* = 0.21. Greater test anxiety in ADHD has been reported in prior studies (Nelson et al., 2014; Dan and Raz, 2015), and we measured it as a possible confounding variable. However, in our sample it did not differ between groups.

An examination of the responses to the study habits measure indicated few differences between participant groups. The two groups did not differ in their use of spacing or cramming, the extent to which they re-read textbooks or notes, or time of day during which they studied. However, consistent with previous research (Reaser et al., 2007), individuals with ADHD were less likely than controls to use self-testing, *χ^2^*(1) = 9, *p* = 0.003. Whereas all of the control participants reported using self-testing, eight (22%) individuals with ADHD indicated they did not. Among those who did use self-testing, the groups did not differ with respect as to why (they learned more, they used it to self-assess, or they enjoyed it more), all *p*s > 0.23. The two groups did not differ in their responses to any of the other items, all *p*s > 0.08, suggesting that the groups were well matched in terms of their academic skills and learning habits.

### Performance at encoding

#### Encoding accuracy

**Figure 2:**
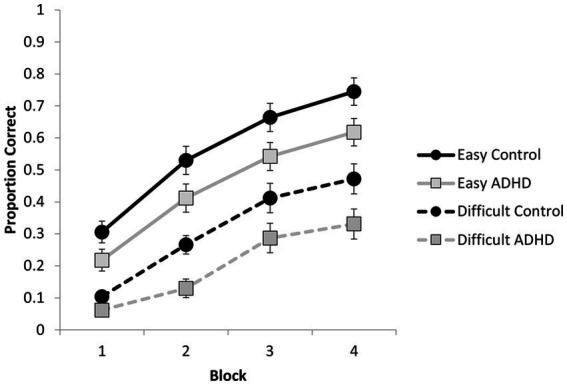
Proportion correct responses during the encoding phase as a function of group, block, and pair difficulty (error bars represent standard error).

In this section, we tested for differences between groups during the encoding phase on the items that were tested and whether any differences emerged as a function of item difficulty and encoding block. We submitted the proportion of correct responses to a 4 (block) × 2 (difficulty) × 2 (participant group) mixed ANOVA, in which block and difficulty were within-subjects factors and group was a between-subjects factor. Overall, participants with ADHD (*M* = 0.33, *SE* = 0.03) performed worse than the cognitively matched control participants (*M* = 0.44, *SE* = 0.03) during the encoding phase, *F*(1, 70) = 5.64, *p* = 0.020, *η_p_^2^* = 0.08. Performance improved across learning blocks, *F*(1.6, 110.33^1^) = 212.56, *p* < 0.001, *η_p_^2^* = 0.75. All pairwise comparisons were significant at the 0.001 level following a Bonferroni correction for multiple comparisons. There was also a main effect of difficulty, *F*(1, 70) = 230.37, *p* < 0.001, *η_p_^2^* = 0.77. All pairwise comparisons were significant, *p*s ≤ 0.001. Pair difficulty and block interacted, *F*(2.09, 146.10) = 6.87, *p* = 0.001, *η_p_^2^* = 0.09. To explore the interaction, we examined the effect of block within each level of difficulty. All effects were significant, all *F*s > 46.5, all *p*s < 0.001, as were all pairwise comparisons (all *p*s ≤ 0.003). The interaction was driven by a steeper increase in retention from Block 1 to Block 2 for easy items compared to difficult items (see Figure 2). No other interactions were significant, all *F*s < 1. Notably, ADHD status did not interact with block or pair difficulty, suggesting that, although this group performed worse than the cognitive control group, both groups were similarly affected by difficulty and showed similar benefits with repeated testing on the word pairs at encoding.

#### Encoding strategies

Participants were asked to describe the encoding strategies they used to learn the word pairs. Participants could report multiple strategies. However, we did not ask for any information about the timing of these strategies (e.g., whether a participant started with one strategy and then switched to another as the learning blocks progressed). These self-reports were independently coded by two coders with an inter-rater reliability measured using the intraclass correlation coefficient (ICC). The ICC was 0.9 indicating excellent agreement between the coders. Responses were coded in terms of the strategies indicated using the following criteria: *Shallow* processing (e.g., focusing on surface features such as sounds or letters shared across the pair items, rote repetition); *intermediate* processing (e.g., combining sound level information with some semantic processing, such as making a sentence with a Swahili word); or *deep* processing (e.g., attending to imagery, meaning, or creating meaningful sentences). Coders also noted the use of the *keyword method* (McDaniel and Pressley, 1984), which is often taught in language courses and involves finding a known word that is a neighbor to the Swahili word and connecting it via mental imagery to the translation equivalent (e.g., *nanga* means anchor, so one might use the keyword *orange* and visualize an orange stuck on an anchor). Proportions of reported strategies are presented in Table 2. Relative to control participants, those with ADHD were significantly less likely to report using deep strategies, *χ^2^*(1) = 5.10, *p* = 0.024, although the total number of strategies and use of shallow strategies did not differ across groups.

**Table 2:** Percentage of participants endorsing a self-reported strategy during the encoding phase as a function of participant group.

	ADHD	Control	*χ^2^*	*p*-value
Shallow	69%	68%	0.01	0.94
Intermediate	17%	11%	0.40	0.53
Deep	30%	57%	5.1	0.024
Keyword	19%	29%	0.81	0.37
Self-testing	3%	6%	0.29	0.59

Because multiple responses were possible, totals are higher than 100%.

### Performance at final test

#### Final performance as a function of group and retrieval practice

**Figure 3:**
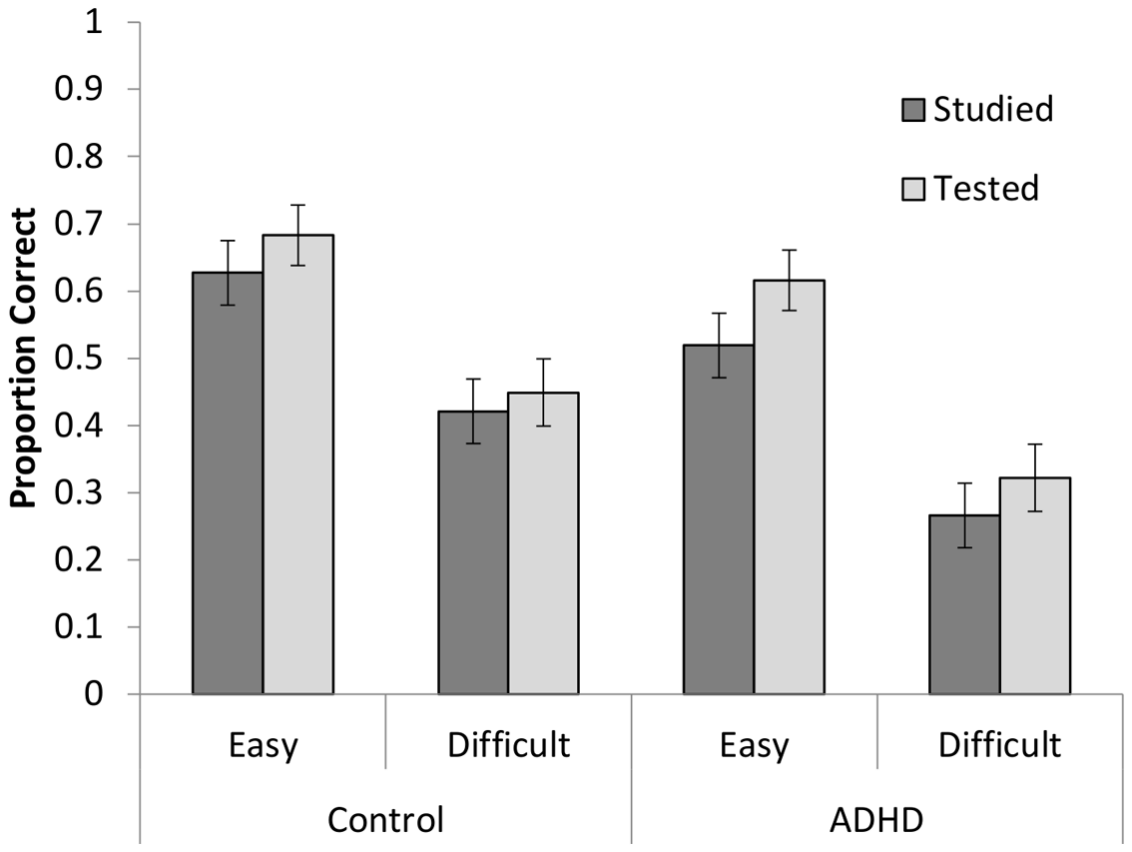
Proportion of correctly recalled items as a function of participant group, encoding condition, and pair difficulty on the final test (errors bars represent standard errors).

In this section we examined whether we would see a difference in the TE between our ADHD participants and cognitive matched controls and crucially whether any group differences in the TE interacted with the difficulty of the word pairs. This was tested by submitting the proportion of correct responses on the final test to a 2 (condition: repeated study vs. repeated test) x 2 (pair difficulty) x 2 (participant group) mixed ANOVA, in which condition and pair difficulty were within-subjects factors and group a between-subjects factor (see Figure 3). Pairs that were repeatedly tested were recalled better (*M* = 0.52, *SE* = 0.03) than pairs that were restudied (*M* = 0.46, *SE* = 0.03), *F*(1, 70) = 10.27, *p* = 0.002, *η_p_^2^* = 0.13, thus demonstrating a TE. Easy pairs were recalled more successfully (*M* = 0.61, *SE* = 0.03) than difficult pairs (*M* = 0.37, *SE* = 0.03), *F*(1, 70) = 265.67, *p* < 0.001, *η_p_^2^* = 0.79 consistent with the norming data (Nelson and Dunlosky, 1994). No interactions were significant, all *F*s ≤ 3.01, *p*s ≥ 0.08. Thus, both participant groups appeared to benefit similarly from repeated testing relative to repeated study (*cf.* Knouse et al., 2016, 2020).

#### Final performance as a function of retrieval success

Retrieval success has been shown to moderate the magnitude of the TE with final test performance higher for items correctly retrieved more often during the encoding phase (Butler, 2010). We conducted a conditional analysis to examine whether there were group differences in the extent to which the number of successful retrievals affected performance at final test and whether that varied by item difficulty. Final test performance improved as a function of retrieval success during encoding, *F*(4, 19) = 4946.19, *p* < 0.001, *η_p_^2^* = 0.53, as final recall increased from 0.07 proportion correct for items never recalled during the encoding phase to 0.56 for items recalled once, to 0.78 for items recalled twice, to 0.90 for items recalled three times, and to 0.97 for items recalled four times. There was no main effect of group, *F*(1, 19) = 1.98, *p* = 0.16, or of difficulty, *F*(1, 19) = 3.56, *p* = 0.06, and no interactions, all *F*s < 1.2. In sum, both groups’ final test performance was similarly improved as a function of initial retrieval success.

#### Final performance as a function of encoding strategy

Although our groups were matched on fluid intelligence and working memory capacity, we did see a difference in self-reported encoding strategies with non-ADHD participants reporting significantly greater use of deep encoding strategies. Deeper encoding strategies have long been shown to improve memory performance (Craik and Tulving, 1975; Coane, 2013). We examined the extent to which self-reported strategy, shallow or deep, predicted final memory performance. The overall regression model was significant, *F*(2,70) = 11.6, *p* < 0.001 *R^2^* = 0.25, with deep strategy use significantly predicting performance, *t* = 4.80, *p* < 0.001, whereas the use of shallow strategies did not, *t* = 1.05, *p* = 0.30. We then reexamined the group difference in memory performance at encoding including deep strategy use as a covariate and found the effect of group was no longer significant at encoding, *F*(1, 69) = 1.97, *p* = 0.17, *η_p_^2^* = 0.03. This suggests that the encoding differences found between the students with and without ADHD may be due to differential use of deep strategies.

### Effects of medication status in ADHD participants at encoding and final test

**Figure 4:**
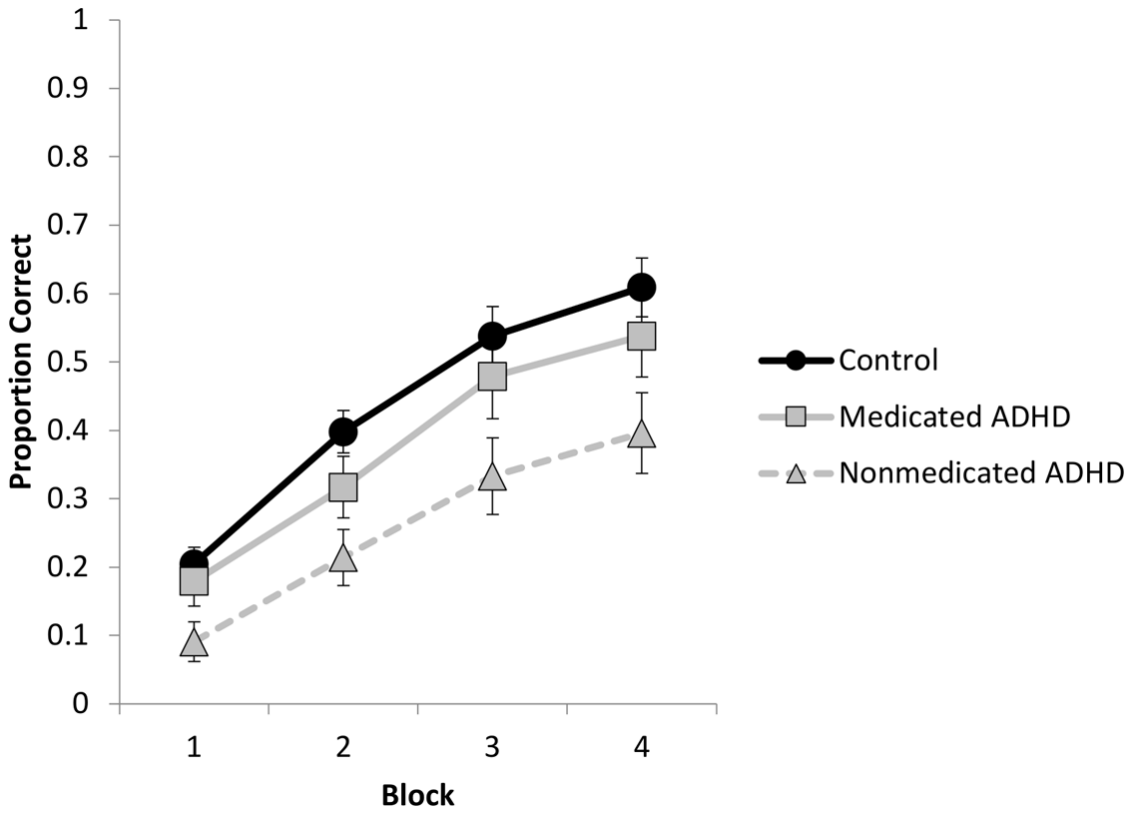
Proportion correct responses during the encoding phase as a function of group (control, medicated ADHD, nonmedicated ADHD) and block (error bars represent standard error).

As we reported in the Participants section, 20 out of 36 participants with ADHD reported taking medication for their ADHD diagnosis at the time of the study. Given research suggesting that ADHD medication can improve memory performance (Advokat and Scheithauer, 2013), we examined whether memory performance differed depending on medication status. We reanalyzed both the encoding and final test data, treating our medicated (*n* = 20) and unmedicated (*n* = 16) participants with ADHD now as separate groups compared to the control group. Group assignment for participants with ADHD was based on whether they reported taking medication on the days of the study. A comparison of self-reported symptomatology between our medicated and unmedicated groups was not significant, *p* < 0.99. As in our initial analysis of encoding performance, there were main effects of difficulty, *F*(1, 69) = 236.37, *p* < 0.001, *η_p_^2^* = 0.77, block [*F*(2, 69) = 202.92, *p* < 0.001, *η_p_^2^* = 0.75, and an interaction between difficulty and block, *F =*(1, 69) = 9.04, *p* = 0.004, *η_p_^2^* = 0.12]. The main effect of group was also significant, *F*(2, 69) = 4.54, *p* = 0.014, *η_p_^2^* = 0.12, and did not interact with any other factors. Post-hoc comparisons using a Bonferroni correction revealed a significant difference in performance between the control group and the unmedicated ADHD group, *p* = 0.011, whereas the differences between the medicated ADHD group and the control group (*p* = 0.86) and between the medicated and unmedicated ADHD groups (*p* = 0.23) were not significant. These data are shown in Figure 4. Turning to encoding strategy, we specifically revisited the use of deep strategies and found the unmedicated group had significantly fewer participants reporting deep strategies (2 out of 16) than both the control group (20 out of 36), *χ^2^*(1) = 8.92, *p* = 0.003 and the medicated ADHD group (9 out of 20), *χ^2^*(1) = 4.43, *p* = 0.04, whereas the medicated ADHD group did not differ from the control group, *χ^2^*(1) = 0.75, *p* = 0.39.

**Figure 5:**
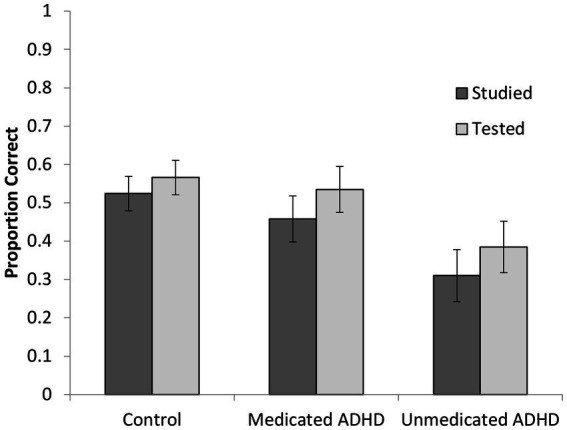
Proportion of correctly recalled items as a function of participant group and encoding condition on the final test (errors bars represent standard errors).

Turning to performance at final test, we reanalyzed the data using a 2 (condition: repeated study vs. repeated test) × 2 (pair difficulty) × 3 (participant group) mixed ANOVA. There were significant main effects of condition and difficulty with no interactions, *F*s < 1. However, now the main effect of group was significant, *F*(2, 69) = 3.26, *p* = 0.04, *η_p_^2^* = 0.09. *Post hoc* comparisons revealed that the difference between the unmedicated ADHD group and the control group was significant (*p* = 0.04), whereas the difference between the two ADHD groups (*p* = 0.27) and between the medicated ADHD group and the control group (*p* > 0.99) were not. These data are shown in Figure 5. Finally, we examined whether there were any differences between our three groups on the cognitive measures used to create the control group as one could hypothesize that unmedicated participants may have lower working memory or fluid intelligence scores which could contribute to memory differences. A one-way ANOVA found no significant group differences in working memory, fluid intelligence or vocabulary, all *p*s > 0.10.

## Discussion

Overall, our results are consistent with the prior literature both in terms of the benefits of retrieval practice for college students with ADHD (Knouse et al., 2016, 2020) and the use of poorer encoding strategies in individuals with ADHD (Andersen et al., 2013; Skodzik et al., 2017). Our data suggest that the benefits of testing do not depend on ADHD status and do not interact with item difficulty or medication use, making retrieval practice a widely applicable strategy to improve all students’ memory performance.

However, our examination of the effects of medication status found unmedicated students performed significantly worse than the control group at both encoding and final test, while medicated students performed similarly to the controls. The unmedicated group also reported significantly less use of deep encoding strategies than both the medicated group and the controls. Although these results are preliminary, they are consistent with work conducted with unmedicated children and adolescents with ADHD that reports poorer memory performance due to less effortful encoding strategies (Egeland et al., 2010; Andersen et al., 2013) and a meta-analysis of long-term memory performance in adults with ADHD finding deficits at encoding, but not retrieval (Skodzik et al., 2017). Our medication analyses are also consistent with research finding improved long-term memory performance with stimulant use (Smith and Farah, 2011; Advokat and Scheithauer, 2013) and studies reporting improved effort and motivation with the use of ADHD medication (Wardle et al., 2011; Addicott et al., 2019).

Relating our study to the prior studies of the testing effect in ADHD, our results are consistent with Knouse et al. (2016, 2020). In addition to replication, our work demonstrated that this remains true even when item difficulty is varied and when participants are cognitively matched on working memory and fluid intelligence. We were also able to demonstrate that the TE does not appear to be affected by medication status (both medicated and unmedicated students showed an equivalent TE) or poor encoding performance. Both Knouse et al. (2016, 2020) had equivalent performance at encoding between their participant groups. The former matched ADHD and control participants on vocabulary and overall memory performance, with ADHD participants performing slightly better at encoding. The latter had high performing ADHD participants, with higher vocabulary scores and better overall performance at final test for the ADHD group over controls in several analyses. Dudukovic et al. (2015) had worse performance at encoding for their participants and this could be one reason for the difference seen in retrieval practice. However, our data do not support that interpretation, as our unmedicated group performed significantly worse at encoding and yet did not differ in the size of the testing effect. In terms of medication status, 78% of Dudukovic’s sample was on medication, as were 50% of Knouse et al.’s (2020) participants. Knouse et al. (2016) assessed medication status in their screening but did not report what percentage was medicated. In their discussion, Knouse et al. (2020) raised the issue of medication and deemed it unlikely that medication status would affect the testing effect, based on prior studies showing effects of medication are on memory encoding, not retrieval. Our data support this assertion.

Another important takeaway from our results is that although retrieval practice is equally effective, it does not make up for the deficits at encoding seen in the unmedicated participants with ADHD. Consistent with prior work showing that the performance deficits in ADHD are largely due to poor encoding, this suggests that, once encoding is successful, retrieval can and does occur. However, although retrieval practice appears to promote better encoding than re-study, it does not compensate for poor encoding strategies. Thus, an emphasis on the remediation of deficits at encoding may be needed for some students with ADHD to perform optimally. Participants using deeper encoding strategies have been shown to outperform those benefiting from retrieval practice (Delaney et al., 2010; Minear et al., 2018). However, there is little research on the training of memory strategies in individuals with ADHD (Jonkman et al., 2016) and some evidence that instructed use of elaborative encoding may not lead to better memory than retrieval practice at least in some populations (Coane, 2013). It is also unclear whether the possible deficits in effort reported in unmedicated ADHD individuals would impact the use of any strategies learned.

## Limitations and future directions

Our results suggest that the significant difference seen during encoding between the ADHD group and control group in our initial analysis may have been due to having a mixture of medicated participants with ADHD who performed similarly to controls and un-medicated participants with ADHD who performed significantly worse than the control group. This is consistent with a number of studies demonstrating improved memory performance with use of stimulant medication (Advokat and Scheithauer, 2013). However, we acknowledge that these findings warrant replication because, in addition to power concerns, we have no information on the type or dosage of medication, the degree to which participant adhered to their medication regimen, and the frequency or recency with which participants took medication. Further, we recognize that our ADHD group was based on self-report of previous diagnosis rather than a multi-informant and multi-modal, evidence-based assessment, which is the gold standard of determining ADHD status (Barkley, 2015).

## Data availability statement

The datasets presented in this study can be found in online repositories. The names of the repository/repositories and accession number(s) can be found at: https://doi.org/10.15786/20520690.v1, University of Wyoming libraries data repository.

## Ethics statement

The studies involving human participants were reviewed and approved by Colby College Institutional Review Board and University of Wyoming Institutional Review Board. The patients/participants provided their written informed consent to participate in this study.

## Author contributions

JC and MM contributed to the concept, stimulus development, data collection, and analyses. LC and SB collected, scored, and coded data. MM, JC, LC, SB, and JS contributed to the writing. All authors contributed to the article and approved the submitted version.

## Funding

The research was supported by a Social Sciences Division Grant from Colby College awarded to JC. At the time of the analyses and writing, JC was supported by a James S. McDonnell Foundation Understanding Human Cognition award # 220020426 and MM was supported by National Science Foundation Grant # 1660996.

## Conflict of interest

The authors declare that the research was conducted in the absence of any commercial or financial relationships that could be construed as a potential conflict of interest.

## Publisher’s note

All claims expressed in this article are solely those of the authors and do not necessarily represent those of their affiliated organizations, or those of the publisher, the editors and the reviewers. Any product that may be evaluated in this article, or claim that may be made by its manufacturer, is not guaranteed or endorsed by the publisher.
